# The Glycosyltransferase Pathway: An Integrated Analysis of the Cell Metabolome

**DOI:** 10.3390/metabo12101006

**Published:** 2022-10-21

**Authors:** Yannick Audet-Delage, Michèle Rouleau, Lyne Villeneuve, Chantal Guillemette

**Affiliations:** 1 Centre Hospitalier Universitaire de Québec Research Center—Université Laval, Faculty of Pharmacy, and Université Laval Cancer Research Center (CRC), R4720, 2705 Blvd Laurier, Québec, QC G1V 4G2, Canada; 2 Canada Research Chair in Pharmacogenomics, Université Laval, Québec, QC G1V 4G2, Canada

**Keywords:** metabolomics, glucuronidation, cell metabolism, glucose, TCA cycle, lipids

## Abstract

Nucleotide sugar-dependent glycosyltransferases (UGTs) are critical to the homeostasis of endogenous metabolites and the detoxification of xenobiotics. Their impact on the cell metabolome remains unknown. Cellular metabolic changes resulting from human UGT expression were profiled by untargeted metabolomics. The abundant UGT1A1 and UGT2B7 were studied as UGT prototypes along with their alternative (alt.) splicing-derived isoforms displaying structural differences. Nineteen biochemical routes were modified, beyond known UGT substrates. Significant variations in glycolysis and pyrimidine pathways, and precursors of the co-substrate UDP-glucuronic acid were observed. Bioactive lipids such as arachidonic acid and endocannabinoids were highly enriched by up to 13.3-fold (*p* < 0.01) in cells expressing the canonical enzymes. Alt. UGT2B7 induced drastic and unique metabolic perturbations, including higher glucose (18-fold) levels and tricarboxylic acid cycle (TCA) cycle metabolites and abrogated the effects of the UGT2B7 canonical enzyme when co-expressed. UGT1A1 proteins promoted the accumulation of branched-chain amino acids (BCAA) and TCA metabolites upstream of the mitochondrial oxoglutarate dehydrogenase complex (OGDC). Alt. UGT1A1 exacerbated these changes, likely through its interaction with the OGDC component oxoglutarate dehydrogenase-like (OGDHL). This study expands the breadth of biochemical pathways associated with UGT expression and establishes extensive connectivity between UGT enzymes, alt. proteins and other metabolic processes.

## 1. Introduction 

Maintenance of cellular homeostasis relies on a variety of biosynthetic and catabolic pathways, and involves plentiful biomolecules. The glycosylation pathway mediated by UDP-glycosyltransferases (UGTs) is one of these key metabolic processes. It regulates the biological activity of diverse biochemical compounds, by inactivating them through conjugation with glucuronic acid (GlcA) or, less frequently, with glucose (Glc), the sugar moieties of the co-substrates UDP-GlcA or UDP-Glc [1,2]. The glycosylation reaction also leads to an increased polarity of the aglycone, promoting the excretion of the conjugated product through bile and urine [1,2]. In humans, 19 UGT1 and UGT2 enzymes are involved in the elimination of drugs from all classes, toxins and other xenobiotics disturbing various cellular processes. UGTs also regulate the bioavailability and activity of diverse endogenous metabolites including the product of heme catabolism bilirubin, sex steroid hormones, signaling lipids and serotonin [1,3,4,5]. For example, a number of receptors are activated by substrates of UGT enzymes, including sex steroids and prostaglandins [3,6,7]. These metabolites are involved in signal transduction pathways that control key transcription factors acting as major regulators of gene expression programs [8,9]. Hence, many of the metabolite substrates of UGTs are known to critically influence gene expression, cell signaling, adhesion, migration, proliferation, immune responses, and other cellular processes [8]. Consequently, UGT expression and activity impact multiple cellular processes [10,11,12]. 

Our knowledge of the (epi)genetic, transcriptional and post-transcriptional regulation of UGT metabolism is constantly expanding [1,9,13,14,15,16]. Alternative splicing has recently emerged as another key process in the regulation of UGT expression and activity [10,12,17,18,19]. A comprehensive quantification of the UGT transcriptome based on high-throughput RNA sequencing identified over 130 diverse novel UGT variants with distinct structural and functional features [12,20]. Based on functional assays, some of these alternate (alt.) UGT isoforms are enzymatically inactive, but inhibit or induce the enzyme activity of canonical UGTs. These alternative variants emerge as novel regulators of the glycosylation pathway, and potentially of additional cellular metabolic pathways [10,11,12]. However, for many alt. UGTs, their function remains unknown, including their potential impact on global cell metabolism. 

Multiple recent reports have highlighted that the expression of UGT enzymes and alternative variants is frequently perturbed in disease conditions and especially in tumors [20,21,22,23,24,25,26]. In fact, metabolic alterations constitute one well documented strategy exploited by cancer cells to sustain rapid cell proliferation and evasion. Remarkably, UGTs constitute the main enzymes of the pentose and glucuronate interconversion (PGI) pathway, one of the most commonly affected metabolic pathways in cancer cells [27]. In addition, several specific UGTs emerge as prognostic markers in multiple cancer types (leukemia, lung, prostate, colon, breast, bladder, etc.) and as predictive markers of drug responses, including in treatment-naïve patients [3,14,21,25,28,29,30,31,32]. However, the impact of UGT enzymes and alt. proteins on the cell metabolome has received little attention.

**Figure 1:**
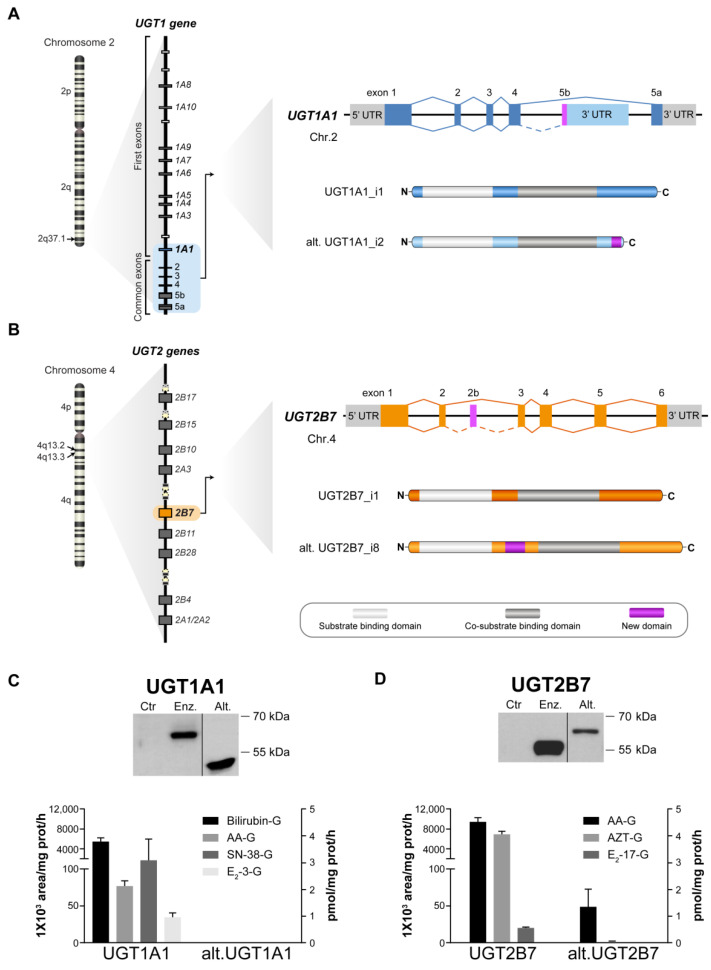
Expression and activity of UGT enzymes and alt. proteins in UGT-negative HEK293 cells. (**A**) UGT1A1 and (**B**) UGT2B7 as prototypical examples of the family UGT1A and UGT2, respectively. The splicing events in the UGT1 and UGT2B genes leading to the UGT1A1 and UGT2B7 canonical enzymes (full lines) and alt. proteins (dashed lines) are schematized. The UGT1A1_i2 alternative protein (alt. UGT1A1) possess a truncated C-terminal, resulting from a stop codon located in the exon 5b, whereas the UGT2B7_i8 alternative protein (alt. UGT2B7) has an in-frame insertion of 32 amino acids encoded by the exon 2b. Stable expression and glucuronidation activity of UGT1A1 (**C**) and UGT2B7 (**D**) enzymes (enz.) or alternative proteins (alt.) in HEK293 cells. Expression was revealed by immunoblotting of microsomal fractions of UGT-expressing cell models and control cells (Ctr). UGT1A1 and UGT2B7 enzymes displayed enzymatic activity toward typical substrates of these isoenzymes. Assays were conducted with microsomal extracts of each cell model incubated with bilirubin, arachidonic acid (AA), estradiol (E2), 7-ethyl-10-hydroxy-camptothecin (SN-38) or zidovudine (AZT). Activities are reported in pmol/mg prot/h (right axis), except for bilirubin and AA (1 × 10^3^ area/mg prot/h; left axis). No activity was detected in Ctr cells (not shown).

We hypothesized that a potential functional interplay between UGTs and other cellular pathways has consequences on the cellular metabolome, possibly beyond the control of metabolites known as UGT substrates. As a primary objective, we examined changes in intracellular metabolite concentrations induced by UGT expression using untargeted metabolomics. We focused on two of the most studied UGT enzymes, the bilirubin-conjugating enzymes UGT1A1 and UGT2B7, that exhibit broad substrate specificity and whose expression is changed in oncogenic settings. We also included alt. UGT isoforms exhibiting major structural differences and novel in-frame sequences, namely the shorter protein UGT1A1 isoform 2—or UGT1A1_i2—with an in-frame C-terminal sequence, and the longer UGT2B7_i8 protein, with an internal in-frame peptide sequence (Figure 1) [17,20]. Because alt. UGT proteins are co-expressed with UGT canonical enzymes in human tissues and were shown to modulate glycosylation activity in cell-based assays, we also tested the impact of co-expressing canonical and alt. UGT proteins on the cell metabolome. Our findings expand our knowledge of biochemical changes associated with UGT expression beyond glycosylation activity and highlight an important connectivity with cell metabolism.

## 2. Materials and Methods

### 2.1. Cell Culture and Metabolomics

HEK293 were cultured in standard conditions, i.e., DMEM medium with 4.5 g/L glucose, 2 mM glutamine, 1 mM sodium pyruvate, 10% fetal bovine serum, as well as 100 IU penicillin and 100 µg/mL streptomycin. Cells stably expressing the UGT1A1 enzyme, its alternative isoform UGT1A1_i2, the UGT2B7 enzyme, its alternative isoform UGT2B7_i8 or the parental plasmid vector as a control were generated as described [12,17]. Protein expression was maintained using appropriate selection antibiotics supplemented to culture media, i.e., G418 (1 mg/mL, Life Technologies Inc., Burlington, ON, Canada) or blasticidin (10 mg/mL, Wisent Bioproducts, St-Bruno, QC, Canada). The following antibodies were used for the detection of UGTs by Western blotting: UGT1A1_i1 RC-71; UGT1A1_i2: 4C5E7; UGT2B7_i1 and UGT2B7_i8: Protein Tech group AP16661-1-AP as described [12].

For metabolomics analyses, cell pellets (40 × 10^6^ cells) were rinsed with 1 mL ice-cold PBS and centrifuged (5 min, 525× *g*), flash-frozen on dry ice, then stored at −80 °C until shipment to Metabolon (Morrisville, NC, USA). Five biological replicates at different passages were prepared for each cell model. Metabolomics profiling was conducted by Metabolon Inc. based on ultra-high-performance liquid chromatography-mass spectrometry (UPLC-MS/MS) [33]. Proteins were removed from samples by methanol-induced precipitation and centrifugation. Each sample extract was divided into five fractions for analyses using four methods: two for analysis by two separate reverse phase (RP)/UPLC-MS/MS methods with positive ion mode electrospray ionization (ESI), one for analysis by RP/UPLC-MS/MS with negative ion mode ESI, one for analysis by hydrophilic interaction chromatography (HILIC) UPLC-MS/MS with negative ion mode ESI, and one sample was reserved for backup. Several types of controls were analyzed in concert with the experimental samples. Each reconstitution solvent contained a series of internal standards at fixed concentrations to ensure injection and chromatographic consistency. Raw data were extracted, peak-identified and QC processed using Metabolon proprietary hardware and software. Compounds were identified through comparison with library entries of purified standards or recurrent unknown entities. Data were normalized for protein concentration, as well as log-transformed and median-scaled to attain variables homoscedasticity. Since four groups were compared by UGT subtype, we selected the analysis of variance (ANOVA). *p*-Values reported throughout the paper were corrected with Tukey’s post hoc test for multiple comparisons, as well as for multiple testing using the false discovery rate (FDR) method. 

### 2.2. UGT Enzymatic Assays

Microsomes (20 µg) were incubated with bilirubin (10 µM; Sigma, Oakville, ON, Canada), estradiol (E2, 100 µM; Steraloid, Newport, RI, USA), zidovudine (AZT, 300 µM; Sigma), 7-ethyl-10-hydroxy-camptothecin (SN-38, 200 µM; obtained as described [13]) or arachidonic acid (AA, 100 µM; Cayman Chemical, Ann Arbor, MI, USA) in assay buffer (50 mM Tris-HCl pH 7.5, 10 mM MgCl_2_, 5 µg/mL pepstatin, 0.5 µg/mL leupeptin, 2 mM UDP-GlcA, 20 µg/mL alamethicin) at 37 °C. Reactions were stopped with methanol, centrifuged (13,000× *g*, 10 min, 4 °C) and store at −20 °C until MS analysis. Glucuronides were quantified by LC-MS/MS and according to published methods for bilirubin [18], E2 [34], AZT [35] and SN-38 [36]. Arachidonic acid glucuronide (AA-G) was detected with an API 6500 (Sciex, Concord, ON, Canada), operated in multiple reaction monitoring (MRM) mode and equipped with a turbo ion-spray source. ESI was performed in negative ion mode. The chromatographic system consisted of a Nexera (Shimadzu Scientific instruments, Inc., Columbia, MD, USA) equipped with a Synergie RP-Hydro 4.0 µm packing material, 100 × 4.6 mm (Phenomenex, Torrance, CA, USA). The mobile phases were water with 1 mM ammonium formate (A) and methanol with 1 mM ammonium formate (B) at a flow rate of 0.9 mL/min. The initial conditions were of 75% B, followed by a linear gradient up to 90% B in 5 min. This concentration was held for 2 min and then re-equilibrated to initial conditions over 3 min. The MRM transition used for analysis was 479.2 → 303.1 *m*/*z*. The resolution used in those methods for Q1 and Q3 was Unit/Unit. HPLC and MS were controlled through Analyst Software (v1.6.1, AB Sciex LP, Concord, ON, Canada).

### 2.3. Fatty Acid Synthesis Assay

Fatty acid synthase (FASN) activity was based on a published protocol [37]. Briefly, HEK293 cells from two confluent 15 cm Petri dishes were scraped and rinsed with ice-cold PBS. After centrifugation (525× *g*, 5 min), cells were resuspended in lysis buffer (50 mM Tris-HCl pH 7.4, 1 mM EDTA, 150 mM NaCl and complete Protease inhibitor [Sigma]). Cell suspensions were homogenized on ice using a microtip sonicator and a dounce homogenizer. Samples were centrifuged (14,000× *g*, 15 min, 4 °C) and supernatants were quantified using a Bradford assay. Assays were conducted with 100 µg of protein in an assay buffer (200 mM potassium phosphate buffer pH 6.6, 1 mM DTT, 1 mM EDTA, 240 µM NADPH and 30 µM acetyl-CoA). Malonyl-CoA was added to a final concentration of 50 µM. NADPH oxidation rate was monitored during 10 min at λ = 340 nm with a TECAN M1000 Pro (Morrisville, TN, USA). 

### 2.4. Gene Expression Analysis

Flash-frozen cell pellets were obtained as above. RNA extraction was carried out using the RNeasy Plus Mini Kit (Qiagen Inc., Toronto, ON, Canada) as per the manufacturer’s protocol. Reverse transcriptase and qPCR were conducted as previously reported [12]. Primer sequences are listed in Table S1. Data were analyzed using the ΔΔCt method [38].

### 2.5. Co-Immunoprecipitation

Co-immunoprecipitations (Co-IPs) to confirm OGDHL partnership with UGT1A1 alternative protein were carried out on cell lysates from HEK293 cells stably expressing UGT1A1_i2-V5-His and in which OGDHL-Myc-DDK (Origene, Rockville, MD, USA) was transfected using Lipofectamine 3000 (Life Technologies Inc.). Co-IPs were conducted as described [39]. Briefly, proteins were crosslinked with 0.125% paraformaldehyde (Sigma) during 10 min at 37 °C. Crosslink reaction was stopped with glycine (125 mM, pH 3.0) for 5 min at room temperature. Cells were rinsed twice with PBS and lysed in 1 mL of lysis buffer (175 mM Tris-HCl pH 7.4, 150 mM NaCl, 1% Igepal [Sigma], 1 mM DTT, complete Protease Inhibitor) for 1 h at 4 °C. Lysates were homogenized using 18G and 20G needles. After centrifugation (6000× *g*, 10 min), the lysates were splitted and mixed with magnetic beads (Life Technologies Inc.) pre-incubated with either goat anti-V5 antibody (Bio-Techne Canada, Oakville, ON, Canada) or goat anti-IgG (R&D Systems, Inc., Minneapolis, MN, USA). After incubation (2 h, 4 °C), beads were rinsed three times with lysis buffer and subjected to SDS-PAGE. Proteins were detected by immunoblotting with mouse anti-V5 (1:20,000, Life Technologies Inc.) and goat anti-Flag M2 (1:10,000, Sigma) antibodies.

## 3. Results

### 3.1. Validation of Catalytic Properties of Ectopically Expressed UGT Enzymes and Alt. Proteins

We studied the metabolic impact of UGT1A1 and UGT2B7 by stably expressing these enzymes in the UGT-negative cell line HEK293 (Figure 1). The influence of alternative UGT proteins was also studied in the same models, expressed alone or in conjunction with their canonical counterpart. These alternative UGTs are representative of truncated (UGT1A1_i2) and extended (UGT2B7_i8) UGT proteins expressed in several human tissues and perturbed in tumors [12,22]. The shorter alt. UGT1A1_i2 lacks the canonical sequence of 99 amino acids comprising the trans-membrane domain and a short cytosolic charged tail (encoded by exon 5a). This sequence is replaced by a truncated C-terminus encoding a unique sequence of 10 amino acids derived from exon 5b (Figure 1A). Its molecular weight corresponds to approximately 45 kDa, as confirmed by Western blotting. The longer alt. UGT2B7_i8 has a unique 32-residue in-frame internal region derived from the novel exon 2b residing at the interface between the N-terminal substrate-binding domain and the C-terminal co-substrate-binding domain, leading to an apparent molecular mass of 62 kDa (Figure 1B).

Immunoblotting and functional assays with known endogenous (bilirubin, estradiol, arachidonic acid) and xenobiotic (anti-cancer agent SN-38 and the anti-viral agent zidovudine) substrates demonstrate the expression of catalytically active UGT enzymes (Figure 1C,D). In these assay conditions and using UDP-GlcA as the co-substrate, enzyme activity was also observed for the UGT2B7_i8 protein whereas glucuronide formation was not detected for UGT1A1_i2 (Figure 1C,D), consistent with previous reports [12,17].

### 3.2. The Cellular Metabolome Is Broadly Affected by the Expression of Canonical and Alt. UGT Proteins

**Figure 2:**
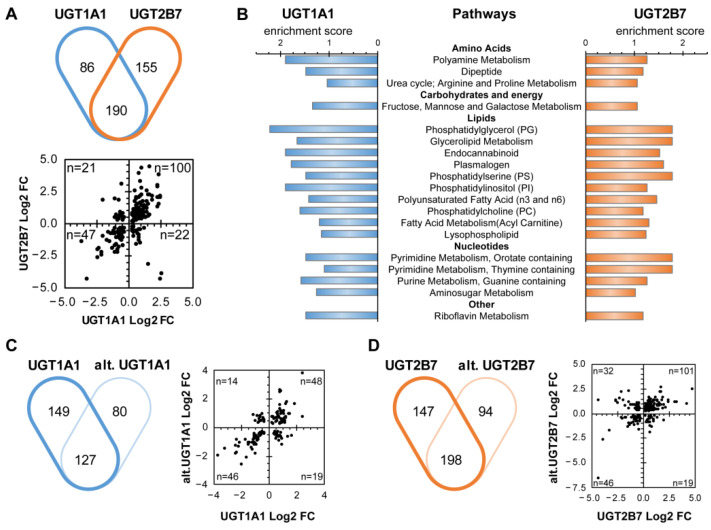
UGT expression induces significant perturbations of metabolite levels. Using an unbiased metabolomic approach, we showed that UGT expression modified intracellular metabolite concentrations in comparison to control cells. (**A**) Common and divergent changes in metabolite levels (*p* < 0.05) for enzyme-expressing models are displayed in the Venn diagram (Upper panel), while the scatter plot (Lower panel) shows the log2 fold change (Log2 FC) of metabolites significantly altered in both cell models. Numbers of metabolites in each quadrant are displayed. (**B**) Pathway enrichment analysis revealed perturbations common to UGT1A1 and UGT2B7. Displayed pathways were enriched in cell models with an enrichment score > 1 and comprised at least 3 metabolites. (**C**) Venn diagram of common and divergent changes in metabolite levels (*p* < 0.05) for UGT1A1 and alt. UGT1A1 models (left). The scatter plot shows the Log2 FC of metabolites altered in both cell models (right). (**D**) Common and divergent changes in metabolite levels (*p* < 0.05) for UGT2B7 and alt. UGT2B7 models are displayed in the Venn diagram (left). The scatter plot shows the log2 FC of metabolites altered in both conditions. Pathway enrichment analysis for (E) and (F) are displayed in Figure S1. Cells were cultured in standard conditions, as described in the Methods. Metabolites were categorized according to Metabolon proprietary database. The complete list of metabolites and their quantification are provided in Tables S2 and S3.

An unbiased metabolomics analysis of cell lysates allowed the detection of 615 metabolites, (Tables S2 and S3). Nearly half of the measured metabolites were significantly changed with the expression of UGT enzymes after correction for Tukey’s post hoc test for multiple comparisons and for FDR. Compared to control cells, the levels of 276 metabolites were significantly altered in cells expressing the UGT1A1 enzyme (176 increased and 100 decreased). The number of perturbed metabolites reached 345 metabolites in cells expressing the UGT2B7 enzyme with 228 increased and 117 decreased (Table 1, Figure 2A). Nearly all measured metabolite classes were perturbed (Table 2; Figure S1). Compared to control cells, levels of glucose, mannose, fructose, along with several glycolytic intermediates, and 2′-deoxycytidine were the most severely changed (by −100 to 18-fold), but dissimilarly across all UGT proteins (detailed below) (Table 2).

UGT1A1 and UGT2B7 canonical enzymes expression affected 19 biochemical routes, including carbohydrate, nucleotide and lipid pathways (Figure 2B). Changes common to UGT1A1 and UGT2B7 enzymes included 190 metabolites, and most notably glucose-6-phosphate, mannose-6-phosphate and arachidonic acid (AA) (Table 2). Alt. UGT proteins also largely affected the cellular metabolome. Alt. UGT1A1_i2 expression modified the levels of 207 cellular metabolites (97 increased and 110 decreased; Table 1, Figure 2C), with 127 metabolites (61%) being also altered in UGT1A1 canonical enzyme-expressing cells. Of those, 94 metabolites (48 increased and 46 decreased) were similarly affected in both cell models, representing 45% and 34% of metabolites altered by alt. and enzyme UGT1A1 expression, respectively. The levels of 292 metabolites (184 increased and 108 decreased) were perturbed by alt. UGT2B7_i8, including 198 metabolites (68%) in common with UGT2B7 enzyme-expressing cells (Figure 2D). Of these, 147 metabolites were similarly modified (101 increased and 46 decreased). The co-expression of enzymes with their alt. variants induced similar numbers of changes relative to each UGT protein expressed alone (Table 1). 

### 3.3. Nucleotide Sugar Precursors Are Significantly Altered by UGT Enzymes and Alt. UGT Proteins

**Figure 3:**
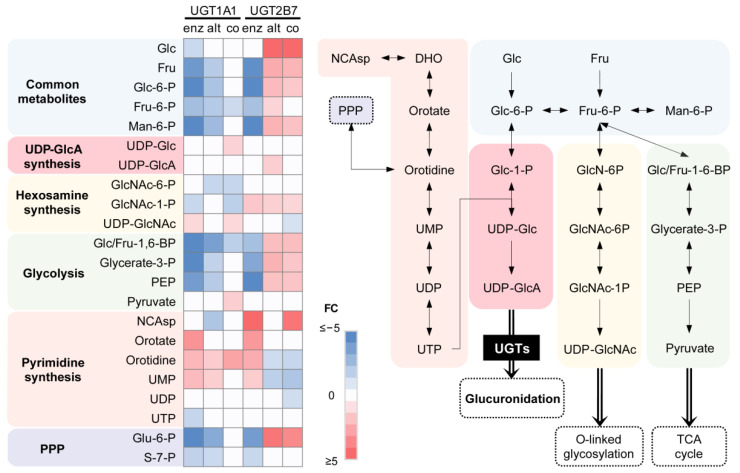
Metabolic precursors of UDP-GlcA synthesis are modified by UGT expression. Several metabolites of the pyrimidine, glycolytic and pentose phosphate pathways are preferentially altered in UGT enzyme-expressing cells. Fold changes (FC) relative to control cells are provided in the heatmap. Non-significant metabolites are displayed in white. Note that glucose and pyruvate were supplemented by the medium. NCAsp, N-carbamoyl aspartate; DHO, dihydroorotate; UMP, uridine monophosphate; UDP, uridine-diphosphate; UTP, uridine triphosphate; Glc, glucose; Glc-6-P, glucose-6-phosphate; Glc-1-P, glucose-1-phosphate; UDP-Glc, UDP-glucose; UDP-GlcA, UDP-glucuronic acid; Fru, fructose; Fru-6-P, fructose-6-phosphate; GlcN-6P, glucosamine-6-phosphate; GlcNAc-6-P, N-acetyl-glucosamine-6-phosphate; GlcNAc-1-P, N-acetyl-glucosamine-1-phosphate; UDP-GlcNAc, UDP-N-acetyl-glucosamine; Man-6-P, mannose-6-phosphate; Glc/Fru-1-6-BP, glucose/fructose-1,6-bisphosphate; PEP, phosphoenolpyruvate; PPP, pentose phosphate pathway.

Consistent with the utilization of UDP-GlcA as a co-substrate by UGT canonical proteins, cellular levels of metabolites related to its synthesis were modified in enzyme-expressing cells. For example, several glycolytic intermediates were reduced in enzyme-expressing cells, while metabolites of the pentose phosphate pathway, as well as purines and pyrimidines, were higher. However, metabolites of the hexosamine pathways remained unaffected (Figure 3). More precisely, the levels of orotate, orotidine and uridine-5′-monophosphate (UMP) metabolites from the pyrimidine synthesis pathway were up to 3.6-fold higher (*p* < 0.01) in UGT enzyme-expressing cells when compared to control cells (Figure 3). Expression of the UGT2B7 enzyme also induced elevated levels of N-carbamoyl aspartate (9.0-fold; *p* = 0.004), a metabolite resulting from a committed step of pyrimidine synthesis. In addition, metabolites from pathways competing with UDP-GlcA synthesis were severely depleted in the presence of UGT enzymes. This included glycolytic and pentose phosphate intermediates such as Glc/Fru-1,6-bisphosphate, glycerate-3-phosphate, phosphoenolpyruvate and 6-phosphogluconate, decreased by −2.6 to −12.5-fold. 

Alt. proteins displayed divergent metabolic alterations relative to respective enzyme-expressing cells, with UGT2B17 isoforms showing the most striking differences. Indeed, glycolytic and pyrimidine synthesis intermediates were elevated in cells expressing the alt. UGT2B7 protein, including the co-substrate UDP-GlcA (1.6-fold; *p* = 0.004) and several glycolytic intermediates (Figure 3). In addition, the alt. UGT2B7_i8 induced a prominent accumulation of glucose (18-fold; *p* < 0.001) and glucose-6-phosphate (2.3-fold; *p* < 0.001), an effect that was not modified by its co-expression with the canonical enzyme. Strikingly, co-expression of alt. UGT2B7 mostly reversed the metabolic impacts of the UGT2B7 enzyme (Figure 3). 

### 3.4. UGT1A1 and UGT2B7 Enzymes Strongly Affect the Lipidome and Lipid Metabolism-Related Gene Expression 

**Figure 4:**
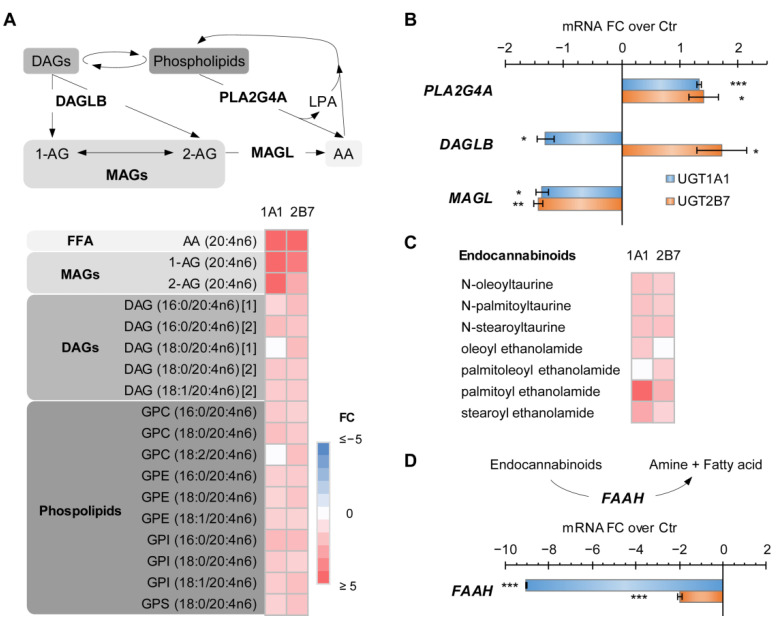
UGT1A1 and UGT2B7 enzymes induce an accumulation of bioactive lipids. (**A**) Arachidonic acid (AA; C20:4n6) and its precursors, 1-arachidonoylglycerol (1-AG) and 2-AG, are elevated in cells expressing UGT1A1 and UGT2B7 enzymes, whereas the levels of these metabolites are almost unaffected by the expression of alt. UGT1A1 and alt. UGT2B7 (Table S2). MAG, monoacylglycerol; DAG, diacylglycerol; GPC, glycerophosphocholine; GPE, glycerophosphoethanolamine; GPI, glycerophosphoinositol; GPS, glycerophosphoserine; LPA, lysophosphatidic acid. MAGL, monoacylglycerol lipase; DAGLB, diacylglycerol lipase B; PLA2G4A, cytosolic phospholipase A2 group IV A. Lipid species are detailed in parentheses. (**B**) The accumulation of AA is linked to an increased expression of *PLA2G4A* and repressed expression of *MAGL* in enzyme-expressing cells, as detected by reverse-transcriptase-quantitative-PCR (RT-qPCR). *DAGLB* expression did not correlate with levels of AA-containing lipids. mRNA fold change (FC) over control (Ctr) cells is displayed. (**C**) Enrichment of endocannabinoids in UGT enzyme-expressing cells. (**D**) The expression of the fatty acid amide hydrolase (FAAH), catalyzing the hydrolysis of endocannabinoids, was decreased in enzyme-expressing cells. Metabolite fold changes (FC) relative to control cells are provided in heatmaps. Non-significant metabolites are displayed in white. *** *p* < 0.001, ** *p* < 0.01, * *p* < 0.05.

Higher arachidonic acid (AA) and AA-containing acyl glycerols was a feature of UGT enzyme-expressing cells, in association with significant transcriptional changes in genes encoding AA metabolic enzymes. Indeed, cell models expressing UGT1A1 and UGT2B7 enzymes showed an accumulation of the bioactive lipid AA, with levels increased by 5.4 and 13.3-fold (*p* < 0.001) in UGT1A1 and UGT2B7 cells, respectively, when compared to control cells (Figure 2A and Figure 4A). Numerous poly-unsaturated fatty acids (PUFAs) and endocannabinoids were also significantly enriched in UGT enzyme-expressing cells when compared to control cells, an effect that was abrogated by co-expression with their alt. isoforms (Table S2). Notably, the monoacylglycerol (MAG) 2-arachidonoylglycerol (2-AG) was significantly higher in UGT1A1 and UGT2B7 enzyme-expressing cells by 5.5 and 2.8-fold (*p* < 0.001), respectively (Figure 4A). In line, increased mRNA expression of PLA2G4A, encoding an enzyme releasing AA from phospholipids, and lower expression of MAGL, coding for an enzyme catalyzing MAG hydrolysis, were observed in both UGT enzyme-expressing cells (Figure 4B). In addition to 2-AG, other endocannabinoid molecules also accumulated in enzyme-expressing cells (Figure 4C). This was also consistent with a significantly decreased expression of FAAH, which encodes the fatty acid amide hydrolase, the main enzyme catabolizing these bioactive lipids (Figure 4D). 

**Figure 5:**
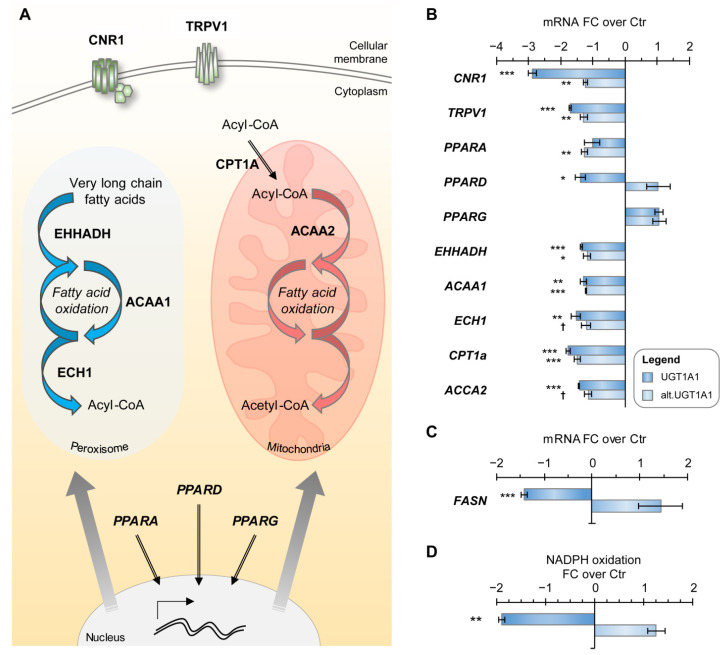
The cannabinoid system is downregulated in cells expressing UGT1A1 enzyme and its alt. protein. (**A**) Scheme of the endocannabinoid system in HEK293 cells, including cannabinoid receptors (CNR1, TRPV1), peroxisome proliferator-activated receptors (PPARs) and some PPAR targets. (**B**) Gene expression of the endocannabinoid system in HEK293 cells expressing UGT1A1 enzyme and alt. UGT1A1. Expression of most genes is perturbed in both cell models and preferentially reduced in UGT1A1 enzyme-expressing cells. Cells expressing the UGT1A1 enzyme have lower fatty-acid synthase (FASN) (**C**) expression and (**D**) activity than control cells, as detected by an assay measuring NADPH oxidation upon malonyl-CoA addition. EHHADH, Enoyl-CoA Hydratase And 3-Hydroxyacyl CoA Dehydrogenase; ACAA, Acetyl-CoA Acyltransferase; ECH1, Enoyl-CoA Hydratase 1; CPT1A, Carnitine Palmitoyltransferase 1A. *** *p* < 0.001, ** *p* < 0.01, * *p* < 0.05, † *p* < 0.1.

For UGT1A1-expressing cells, we further observed a decreased expression of genes encoding cannabinoid receptors, including the G-protein coupled receptor CNR1 (−2.9-fold; *p* < 0.001) and the transient receptor potential cation channel TRPV1 (−1.7-fold; *p* < 0.001) (Figure 5A,B). Downstream targets of the cannabinoid system were also decreased, such as mRNA expression (−1.4-fold, *p* < 0.001) and activity (−1.9-fold, *p* < 0.01) of the fatty acid synthase (FASN) enzyme, determined by an in vitro assay measuring the oxidation rate of NADPH upon addition of malonyl-CoA (Figure 5C,D). A decreased expression of endocannabinoid-targeted nuclear receptor *PPARD* was observed with no significant modifications for *PPARA* and *PPARG*. We also perceived a modest but constant decrease in the expression of genes encoding PPAR-regulated mitochondrial and peroxisomal enzymes (Figure 5A,B), suggesting that the activity of these nuclear receptors was repressed. This change in gene expression was more pronounced in cells expressing the UGT1A1 enzyme, as it remained near control levels in cells expressing the alt. UGT1A1 (Figure 5B).

### 3.5. UGT2B7 Isoforms Affect the Methionine-Creatinine Pathway

**Figure 6:**
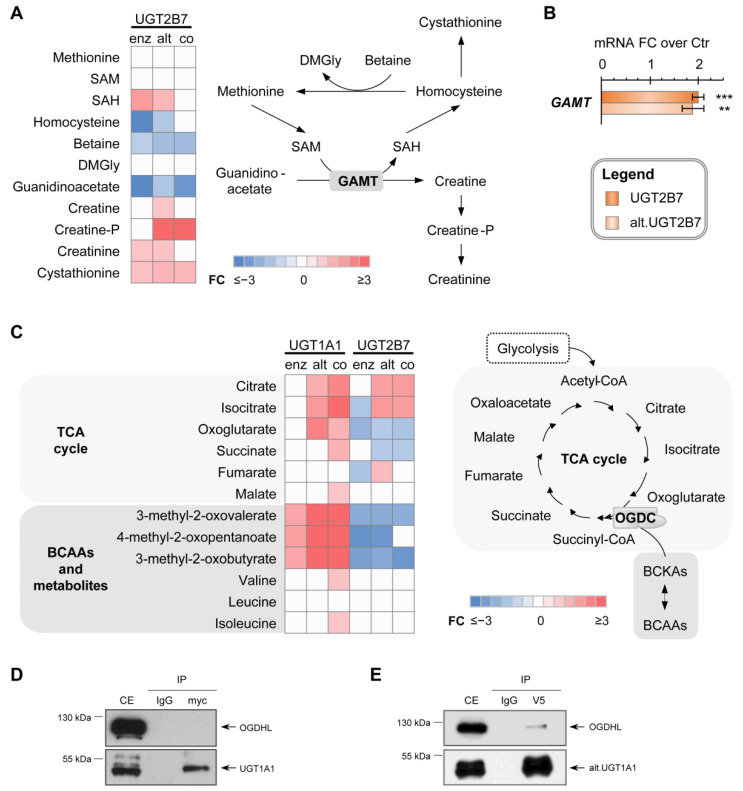
Specific metabolic changes induced by UGT expression. (**A**) Expression of UGT2B7 proteins is associated with elevated metabolites of the creatine pathway. Fold changes (FC) relative to control cells are provided in the heatmap. Non-significant metabolites are displayed in white. SAM, S-adenosyl-methionine; SAH, S-adenosyl-homocysteine; DMGly, dimethyl-glycine; GAMT, guanidinoacetate methyltransferase. (**B**) *GAMT* mRNA expression are increased in cells expressing UGT2B7 proteins when compared to control cells. *** *p* < 0.001, ** *p* < 0.01 (**C**) Metabolites upstream from the oxoglutarate dehydrogenase complex (OGDC) in the TCA cycle are elevated in cells expressing UGT1A1 proteins. Branched-chain keto acids (BCKA) are also enriched in alt. UGT1A1 cells. (**D**) Co-immunoprecipitations (co-IPs) revealed no interaction between UGT1A1 and the oxoglutarate dehydrogenase-like (OGDHL) protein, a key regulator of the OGDC. (**E**) The alt. UGT1A1 interacts with OGDHL, as detected by co-IP. Co-IPs were conducted using anti-myc (UGT1A1) or anti-V5 (alt. UGT1A1) and control IgG antibodies. Western blots were revealed with in-house anti-UGT1A (RC-71; for UGT1A1), anti-V5 (for alt. UGT1A1) or anti-Flag (for OGDHL) antibodies.

For UGT2B7-expressing cells, specific metabolic changes were connected to the creatine pathway. Levels of guanidinoacetate, a metabolite resulting from a committed step of creatine synthesis, were depleted by −3.8 and −1.4-fold (*p* < 0.05) in cells expressing the enzyme and the alt. UGT2B7 protein, respectively (Figure 6A). Supporting the induction of this pathway in UGT2B7-expressing cell models, levels of the downstream metabolite creatinine were 1.2-fold higher (*p* < 0.01) in both cell models when compared to control. Elevations of creatine and creatine-P were also noted in cells expressing alt. UGT2B7 alone or together with UGT2B7 enzyme. The production of creatine from guanidinoacetate requires the simultaneous transformation of S-adenosylmethionine (SAM) into S-adenosylhomocysteine (SAH) by guanidinoacetate N-methyltransferase (GAMT). Supporting an increased GAMT activity, SAH levels were enriched by up to 2.0-fold (*p* < 0.05) in UGT2B7-expressing cells. This is supported by an elevated expression of *GAMT* by up to 2.0-fold (*p* < 0.05) in these cells (Figure 6B). In contrast, few of these metabolites were significantly modified in cells expressing UGT1A1 proteins (Table S2).

### 3.6. The Metabolic Profile of Alt. UGT Support a Functional Protein–Protein Interaction Leading to Altered Mitochondrial Metabolism 

Mitochondrial branched-chain keto acids, i.e., 3-methyl-2-oxovalerate, 4-methyl-2-oxopentanoate and 3-methyl-2-oxobutyrate, derived from branched-chain amino acids (BCAA), were more abundant in alt. UGT1A1 expressing cells (alone or co-expressed with the enzyme) by up to 5.9-fold (*p* < 0.001) when compared to control cells. TCA cycle metabolites, namely citrate, isocitrate and oxoglutarate, were also higher in alt. UGT1A1 expressing cells (by 1.6 to 2.5-fold; *p* < 0.01; Figure 6C). These changes were exacerbated in cells co-expressing canonical and alt. UGT1A1 proteins, whereas they were not observed in cells expressing the UGT1A1 enzyme alone. By contrast, UGT2B7 enzyme expression led to their depletion, suggesting a differential effect of the UGT proteins on mitochondrial metabolism. 

Altered BCAA and TCA cycle metabolites are located upstream of the oxoglutarate dehydrogenase complex (OGDC), among which protein partners of the UGT1As were previously identified by untargeted proteomics experiments in human tissues [10]. While no interaction between the UGT1A1 enzyme and the OGDC component oxoglutarate dehydrogenase-like (OGDHL) protein was detected (Figure 6D), we observed a protein–protein interaction between the alt. UGT1A1 and OGDHL, demonstrated in cell models by immunoprecipitation (Figure 6D,E). This supports the possibility that metabolic changes might be caused by a functional interaction between this member of the OGDC complex and the alt. UGT1A1 protein, explaining the prominent changes of BCAA and TCA cycle metabolites—also linked to FA catabolism—observed in alt. UGT1A1 expressing cells (expressed alone or together with the canonical enzyme). 

## 4. Discussion 

Our study reveals that UGT protein expression triggered significant changes in the cellular metabolome, affecting levels of metabolites in each measured macromolecular group, beyond known UGT substrates. Major changes were observed for both UGT canonical enzymes and their alt. proteins and comprised alterations in carbohydrates, nucleotides and bioactive lipids pathways. Notably, the expression of UGT enzymes induced significant modifications in the metabolism of pyrimidines and glycolysis, suggesting a diversion of these intermediates to support the synthesis of the co-substrates UDP-GlcA and UDP-Glc. Previous lines of evidence implied a limited availability of UDP-GlcA when cells were exposed to important amounts of UGT substrates [40,41,42,43]. An increased pyrimidine metabolism may also reflect a metabolic rewiring to repress UGT activity, as nucleotides represent endogenous allosteric inhibitors [44,45]. Certain UGT enzymes, including UGT2B7, are capable of using UDP-Glc as a co-substrate [46,47,48]. Coherent with a potential increased usage of downstream-related metabolites such as UDP-Glc, depletion in early glycolytic intermediates (e.g., mannose-6-phosphate and glucose-6-phosphate) was observed in canonical UGT2B7 enzyme-expressing cells. Our findings further suggest that UGT enzymes may influence other cellular pathways in which UDP-sugars also participate, including the synthesis of the extracellular matrix component hyaluronan, protein glycosylation, and as ligands of the purinergic receptor P2Y14, which is involved in inflammation, asthma, fibrosis and acute kidney diseases [6,7,49,50]. 

Notable biochemical routes affected by UGT expression were related to lipid pathways, including the bioactive lipid AA and endocannabinoids. The expression of UGT1A1 and UGT2B7 canonical enzymes induced a significant cellular accumulation of AA levels. This was unanticipated given that AA is a UGT substrate for conjugation [5], and that higher UGT expression would be expected to reduce substrate levels. The accumulation of AA within cells may involve a regulatory feedback loop, as suggested by a previous study reporting the repression of UGT1A1 expression by AA supplementation in the hepatic model HepG2, which endogenously expresses this isoenzyme [51]. It may also engage PPAR signaling, as the lower expression of several peroxisomal and mitochondrial PPAR targets involved in mitochondrial fatty acid beta-oxidation was observed in UGT1A1 enzyme-expressing cells. The interplay between UGT enzymes and lipid homeostasis is also supported by the perturbed accumulation of lipid droplets induced by the expression of UGT1A9 and UGT2B7 in HEK293, breast and pancreas cancer cell models [12,52]. Lipid droplets constitute important storage of energy-rich fatty acids and bioactive lipids that also contribute to limiting lipotoxicity [53,54]. Another report further supports the key role of UGT enzymes in maintaining lipid homeostasis, with an effect on the proliferation of cancer cells [52]. An impact of UGT on gene expression and cell metabolism was also observed in a *Ugt1* liver-knock-out mouse model. The loss of Ugt1 functionality in the mouse liver resulted in significant alterations in the expression of several genes including those linked to hormones and fatty acids pathways and pyrimidine metabolism [55]. Accordingly, the interconnection between lipid metabolism and the UGT pathway seems more complex than being related to substrates for these enzymes and likely involves signaling events and modifications of gene expression triggered by bioactive lipids, as well as protein–protein interactions [10,39]. 

Our study demonstrates that this may also be the case for alt. UGT proteins. For instance, we revealed a protein–protein interaction between the alt. UGT1A1 protein and OGDHL, a component of the OGDC enzyme complex and a key control point in the citric acid cycle often bypassed in cancer cells [56,57,58,59,60]. It is plausible that this partnership may explain, at least in part, changes in the levels of TCA cycle intermediates and BCAA metabolites observed in cells overexpressing the alt. UG1A1 protein. Consistent with this notion, our previous work using an alt. UGT1A-depleted cancer cell model showed a shift in energy metabolism, increasing cell dependency on glucose at the expense of oxidative phosphorylation, likely dependent on a functional interaction of UGT1A proteins with the pyruvate kinase M2 (PKM2) enzyme [10]. These protein partners of the alt. UGT1A1 protein are key regulators of energy metabolism, which could be linked to the capacity of UGTs to induce a redirection of carbon skeletons as discussed above. In fact, when compared to UGT1A1 enzyme-expressing cells, those expressing the alt. UGT1A1 displayed similar alterations for several abovementioned metabolic pathways (glycolysis, pyrimidine synthesis and bioactive lipid metabolism), and could be instigated by common protein interactors [10,11,39]. By these interactions, we also exposed that alt. UGT1A proteins, but not UGT1A enzymes, interfere with oligomeric complex formation necessary for scavenging activity of catalase and peroxiredoxin [11]. Expression of the structurally divergent alt. UGT2B7_i8, yet enzymatically active, caused distinct metabolic profiles for several pathways, notably for glycolytic intermediates and nucleotide sugar precursors, which could also result from protein partners interacting with its unique peptide sequence and/or distinct substrate specificity compared to the canonical UGT2B7 enzyme. 

Isoform-specific metabolic and phenotypic changes are likely induced by their divergent primary structure, catalytic function, protein partners and/or subcellular localisation [20]. In line, we previously observed that in contrast to the canonical enzyme, the expression of the alt. UGT2B7_i8 increased cellular adhesion while reducing proliferation, supporting a distinct role for alt. proteins on cellular metabolism [12]. This is also supported by the observation that alt. UGT2B7_i8 expression abrogated many metabolic changes induced by the UGT2B7 enzyme when co-expressed in HEK cells, such as the rewiring of glycolytic intermediates. By contrast, the co-expression of alt. truncated UGT1A1_i2 with its enzyme had pathway-specific effects, such as an exacerbation of the impact of UGT1A1 enzyme on the TCA cycle and repression of its effect on PUFA and MAG. Given their ability to form protein complexes with UGT enzymes [61,62], UGT alt. proteins have the potential to contribute significantly to metabolic changes and those associated with cancer considering their frequent co-expression in cancer tissues. In line, differential isoform usage (isoform switching) is a frequent event in cancer cells, including switches favoring alt. UGT1A1 and UGT2B7 expression that were recently reported in esophageal cancer tissues [26,63].

Because most available immortalized human cancer cell lines express multiple UGTs including enzymes and several forms of alt. UGTs, we selected to conduct this study in the human HEK293 embryonic kidney cell model in which the UGT pathway is inactive due to the lack of endogenous UGT expression (Figure 1) [62,64,65]. Although this represents a limitation of the study, it permitted outlining the specific effects of individual UGT enzymes and alt. proteins on cellular metabolome. Other limitations include the fact that known signalling molecules inactivated by UGTs are found potentially below quantification in cell models or others may not have been part of the metabolite panel detected by this platform. Moreover, relative metabolite quantification hinders our capacity to compare metabolite levels with other studies, an inherent limitation of untargeted metabolomics analyses. However, our profiling highlighted pathways unsuspected to be related to UGTs, including endocannabinoids. It also revealed direct (substrate for conjugation) and indirect (perturbed gene expression) influences of UGTs on bioactive lipids such as AA. This interplay is also supported by the dependency of UGT activity on interactions with membrane phospholipids [66]. Future work will aim at integrating gene expression, enzyme activity and metabolic perturbations to fully appreciate the connectivity between UGT and other metabolic pathways.

Our study unveiled unprecedented and distinctive changes in intracellular metabolites caused by the expression of two major hepatic canonical UGT enzymes and their prototypical truncated and extended alt. proteins with novel in-frame sequences. Data support that the UGT proteins are involved in a regulatory process used by cells to control the activity of their metabolic networks, with broad consequences on cell metabolite levels linking UGTs to novel metabolic pathways and potential biological functions. 

## Figures and Tables

**Table 1:** Overview of metabolic changes associated with UGT protein expression.

	UGT1A1		
Number of Metabolites ^1^	Enzyme	Alternative Isoform	Enz. + Alt.
Total	276	207	241
Up|Down	176|100	97|110	155|86
	UGT2B7		
Number of Metabolites ^1^	Enzyme	Alternative Isoform	Enz. + Alt.
Total	345	292	280
Up|Down	228|117	184|108	149|131

^1^ Number of significantly changed metabolites (*p* ≤ 0.05) relative to control cells. A total of 615 metabolites were measured in the untargeted metabolomics assay. Red indicates increased levels and blue decreased levels. Detailed quantification of metabolites is provided in Tables S2 and S3.

**Table 2:** Top modulated metabolites in UGT-expressing cells.

Cell Line	Pathway	Metabolite	Fold Change	*p*-Value
UGT1A1	Glycolysis, Gluconeogenesis, and Pyruvate Metabolism	Isobar: fructose 1,6-diphosphate, glucose 1,6-diphosphate, myo-inositol 1,4 or 1,3-diphosphate	−12.5	4.4 × 10^−8^
	Fructose, Mannose and Galactose Metabolism	mannose-6-phosphate	−9.1	8.6 × 10^−5^
	Glycolysis, Gluconeogenesis, and Pyruvate Metabolism	glucose 6-phosphate	−5.3	9.4 × 10^−5^
	Pentose Phosphate Pathway	6-phosphogluconate	−5.3	5.8 × 10^−5^
	Glycolysis, Gluconeogenesis, and Pyruvate Metabolism	3-phosphoglycerate	−5.0	2.0 × 10^−6^
	Glycolysis, Gluconeogenesis, and Pyruvate Metabolism	dihydroxyacetone phosphate (DHAP)	−5.0	5.8 × 10^−8^
	Glycerolipid Metabolism	glycerophosphoglycerol	5.4	1.4 × 10^−10^
	Polyunsaturated Fatty Acid (n3 and n6)	arachidonate (20:4n6)	5.4	2.7 × 10^−8^
	Monoacylglycerol	1-arachidonylglycerol (20:4)	5.5	5.9 × 10^−8^
	Purine Metabolism, Adenine containing	adenine	5.5	2.4 × 10^−10^
	Pyrimidine Metabolism, Cytidine containing	2′-deoxycytidine	5.6	9.8 × 10^−6^
	Polyamine Metabolism	N1,N12-diacetylspermine	5.9	1.0 × 10^−4^
UGT2B7	Fructose, Mannose and Galactose Metabolism	mannose-6-phosphate	−20.0	5.9 × 10^−11^
	Pyrimidine Metabolism, Cytidine containing	2′-deoxycytidine	−20.0	5.1 × 10^−10^
	Polyamine Metabolism	N1,N12-diacetylspermine	−14.3	2.2 × 10^−6^
	Polyamine Metabolism	N(1)-acetylspermine	−9.1	1.1 × 10^−7^
	Glycolysis, Gluconeogenesis, and Pyruvate Metabolism	glucose 6-phosphate	−6.3	3.9 × 10^−7^
	Nicotinate and Nicotinamide Metabolism	adenosine 5′-diphosphoribose (ADP-ribose)	−6.3	4.1 × 10^−6^
	Polyunsaturated Fatty Acid (n3 and n6)	arachidonate (20:4n6)	13.3	6.5 × 10^−9^
	Glutathione Metabolism	cysteine-glutathione disulfide	14.5	8.3 × 10^−7^
	Pyrimidine Metabolism, Thymine containing	5,6-dihydrothymine	19.5	3.6 × 10^−9^
	Methionine, Cysteine, SAM and Taurine Metabolism	cystine	21.0	1.4 × 10^−9^
	Pyrimidine Metabolism, Thymine containing	thymine	26.1	7.3 × 10^−9^
	Pyrimidine Metabolism, Uracil containing	uracil	28.3	1.9 × 10^−8^
alt. UGT1A1	Plasmalogen	1-(1-enyl-stearoyl)-2-linoleoyl-GPE (P-18:0/18:2)	−6.3	1.1 × 10^−6^
	Polyamine Metabolism	putrescine	−4.3	8.8 × 10^−8^
	Glycolysis, Gluconeogenesis, and Pyruvate Metabolism	Isobar: fructose 1,6-diphosphate, glucose 1,6-diphosphate, myo-inositol 1,4 or 1,3-diphosphate	−4.0	7.4 × 10^−5^
	Pentose Phosphate Pathway	6-phosphogluconate	−3.6	8.0 × 10^−4^
	Polyamine Metabolism	N-acetylputrescine	−3.6	9.1 × 10^−7^
	Glycolysis, Gluconeogenesis, and Pyruvate Metabolism	dihydroxyacetone phosphate (DHAP)	−3.4	1.7 × 10^−6^
	Leucine, Isoleucine and Valine Metabolism	3-methyl-2-oxovalerate	5.5	7.3 × 10^−7^
	Leucine, Isoleucine and Valine Metabolism	4-methyl-2-oxopentanoate	5.8	2.1 × 10^−6^
	Leucine, Isoleucine and Valine Metabolism	3-methyl-2-oxobutyrate	5.9	1.6 × 10^−6^
	Diacylglycerol	palmitoleoyl-oleoyl-glycerol (16:1/18:1) [1]	6.9	4.9 × 10^−5^
	Diacylglycerol	diacylglycerol (12:0/18:1, 14:0/16:1, 16:0/14:1) [1]	8.0	5.7 × 10^−7^
	Pyrimidine Metabolism, Cytidine containing	2′-deoxycytidine	13.5	1.1 × 10^−7^
alt. UGT2B7	Pyrimidine Metabolism, Cytidine containing	2′-deoxycytidine	−100.0	1.0 × 10^−13^
	Diacylglycerol	diacylglycerol (12:0/18:1, 14:0/16:1, 16:0/14:1) [1]	−7.7	2.2 × 10^−3^
	Pyrimidine Metabolism, Cytidine containing	cytidine	−7.1	5.7 × 10^−7^
	Pyrimidine Metabolism, Cytidine containing	5-methylcytidine	−7.1	9.8 × 10^−6^
	Polyamine Metabolism	N1,N12-diacetylspermine	−6.7	2.0 × 10^−4^
	Endocannabinoid	N-oleoyltaurine	−3.8	7.9 × 10^−6^
	Polyunsaturated Fatty Acid (n3 and n6)	mead acid (20:3n9)	4.5	6.4 × 10^−6^
	Pentose Phosphate Pathway	6-phosphogluconate	4.6	1.8 × 10^−7^
	Pyrimidine Metabolism, Thymine containing	3-aminoisobutyrate	4.8	1.1 × 10^−12^
	Pyrimidine Metabolism, Uracil containing	uracil	5.5	3.0 × 10^−4^
	Plasmalogen	1-(1-enyl-stearoyl)-2-linoleoyl-GPE (P-18:0/18:2)	6.1	1.8 × 10^−7^
	Glycolysis, Gluconeogenesis, and Pyruvate Metabolism	glucose	18.2	5.7 × 10^−7^

## Data Availability

Data is contained within the article and Supplementary Material.

## Supplementary Materials

The following supporting information can be downloaded at: https://www.mdpi.com/article/10.3390/metabo12101006/s1, Figure S1: Pathway enrichment analysis of metabolic changes common to (A) UGT1A1 canonical and alt. proteins and (B) UGT2B7 canonical and alt. proteins. Figure S2: Full original images of Western blots, related to Figure 1 and Figure 6. Table S1: Primers and conditions for qPCR analysis. Table S2: Levels of measured metabolites in UGT-expressing cells relative to control. Table S3: Levels of measured metabolites per sample.
