# Supplementary material for: The Glycosyltransferase Pathway: An Integrated Analysis of the Cell Metabolome
Source: Metabolites. 2022 Oct 21;12(10):1006. doi: 10.3390/metabo12101006 (PMC9609030; doi:10.3390/metabo12101006)
Supplement: Supplementary file 1 [file metabolites-12-01006-s001.zip › Supplementary_Metabolites-1956236/Metabolites_SupplInfo_20221017.pdf]

The glycosyltransferase pathway: an integrated analysis of the cell metabolome

Yannick Audet-Delage[1*], Michèle Rouleau[1], Lyne Villeneuve[1], Chantal Guillemette[1,2]

[1]Pharmacogenomics Laboratory, Centre Hospitalier Universitaire (CHU) de Québec Research Center and Faculty of Pharmacy, Laval University, Québec, Canada.
[2]Canada Research Chair in Pharmacogenomics, Laval University, Québec, Canada.
*Current address: Ottawa Institute of Systems Biology and Department of Biochemistry, Microbiology and Immunology, Faculty of Medicine, University of Ottawa, Ottawa

Supplementary Information:

Figure S1: Pathway enrichment analysis of metabolic changes common to A) UGT1A1 canonical and alt. proteins and B) UGT2B7 canonical and alt. proteins

Figure S2: Full original uncropped and unadjusted images of Western blots

Table S1: Primers and conditions for qPCR analysis
Table S2: Levels of measured metabolites in UGT-expressing cells relative to controls
Table S3: Levels of measured metabolites per sample

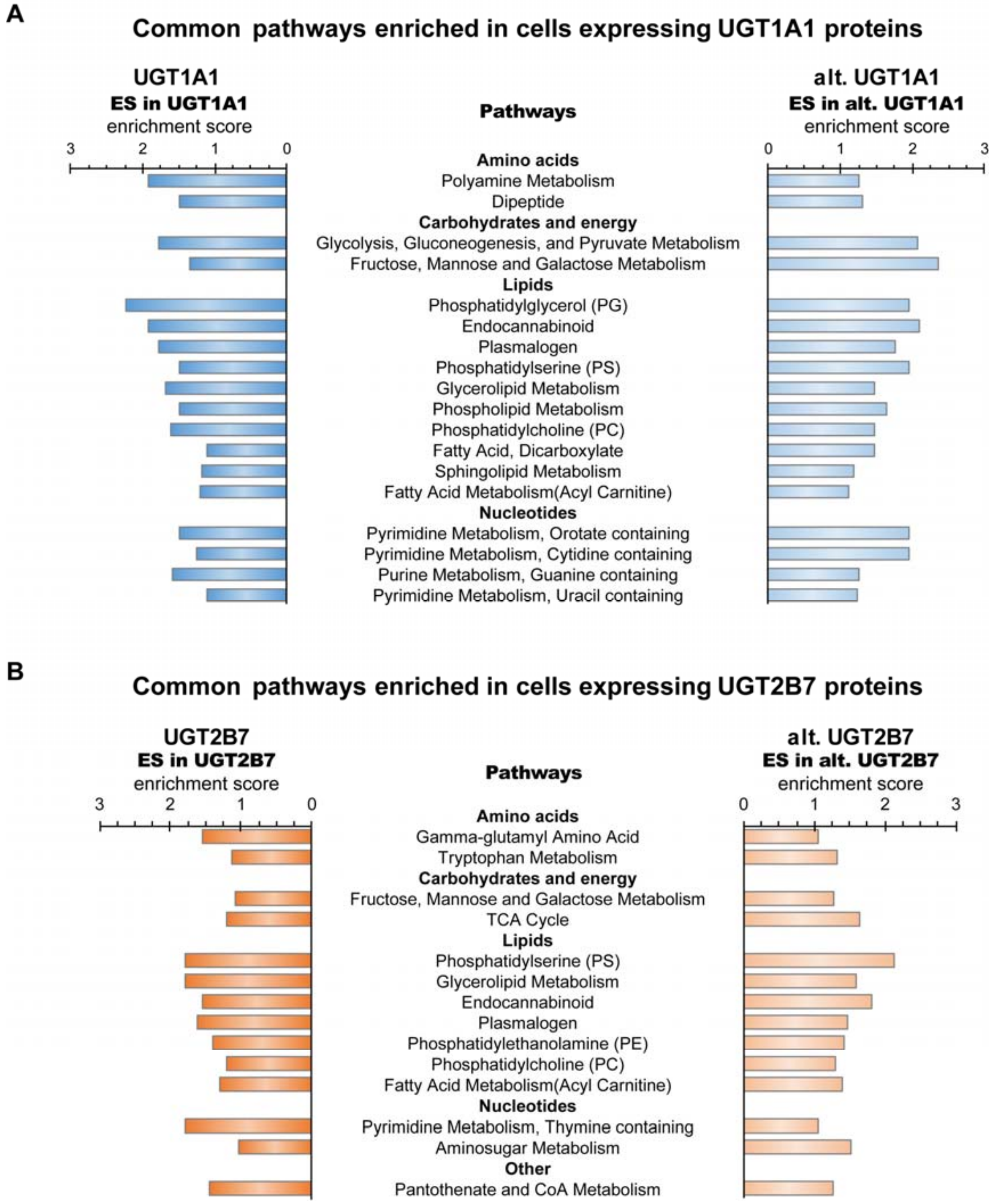

Figure S1. Pathway enrichment analysis of metabolic changes common to A) UGT1A1 canonical and alt. proteins and B) UGT2B7 canonical and alt. proteins. Displayed pathways were enriched in both cell models with an enrichment score > 1 and comprised at least 3 metabolites**.**

A. Related to Figure 1C-D

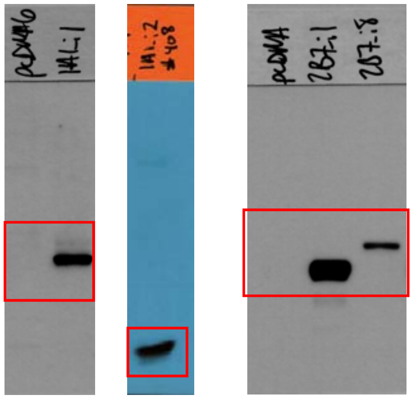

B. Related to Figure 6D-E

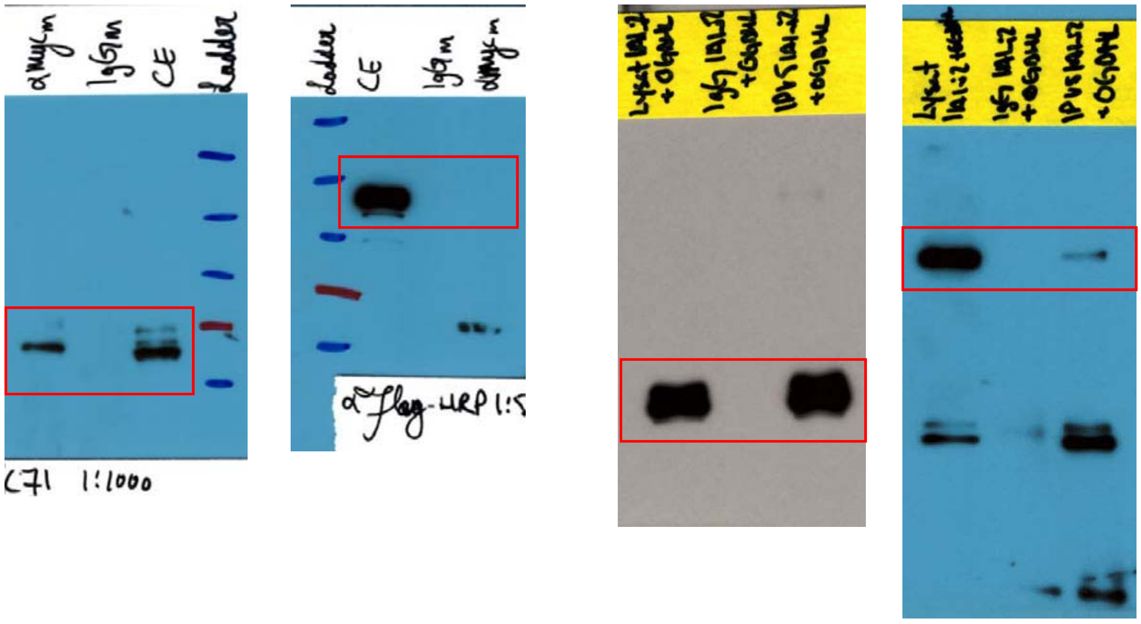

Figure S2. Full original uncropped and unadjusted images of Western blots, related to Fig.1 and Fig.6. Squared regions are shown in A) Figure 1C-D and B) Figure 6D-E.

Table S1. Primers and conditions for qPCR analysis.

Gene	Primer Sequences	Primer concentrations (nM)	Temperature (˚C)
PLA2G4A	F- ACTGCACAATGCCCTTTACC<br>R- CGGGAGCCATAAAAGTACCA	200 nM	60 ˚C
DAGLB	F- TGCTTCATCAGCAACAGGAC<br>R- CAGCAGTCACCACCAATCC	300 nM	60 ˚C
MAGL	F- ATGCAGAAAGACTACCCTGGGC<br>R- TTATTCCGAGAGAGCACGC	200 nM	60 ˚C
FAAH	F- AAGGTGATTTCGTGGACCCC<br>R- CCAGCCGAACGAGACTTCAT	200 nM	60 ˚C
PPARA	F- GCTGGTGTATGACAAGTGCG<br>R- TCTTGGCATTCGTCCAAAACG	300 nM	60 ˚C
PPARD	F- CTTCCAGCAGCTACACAGACCTC<br>R- TACTGGCACTTGTTGCGGTT	300 nM	60 ˚C
PPARG	F- AGATGACAGCGACTTGGCAATA<br>R- AGGGCTTGTAGCAGGTTGTCT	200 nM	60 ˚C
CNR1	F- GATGCGAAGGGATTGCCCC<br>R- GATGGTGCGGAAGGTGGTAT	100 nM	64 ˚C
TRPV1	F- GGGTACACACCTGATGGCAA<br>R- GCCTGAAACTCTGCTTGACC	300 nM	60 ˚C
ACAA1	F- GAGATCAATGAGGCCTTTGC<br>R- GATGCACATGGACACCACTC	300 nM	60 ˚C
ECH1	F- GGGATAGTGGCTTCTCGCAG<br>R- CCAGTGAGGCGAAGGCTAAT	300 nM	60 ˚C
EHHADH	F- GTGAAGGAGCTTGGTGGTGT<br>R- AAAAGAAGTGGGTGCCAATG	200 nM	60 ˚C
CPT1a	F- AGATGAGTCGTGCCACCAAG<br>R- CCACCAGTCGCTCACGTAAT	200 nM	60 ˚C
FASN	F- CTTCAAGGAAGGCGTGA<br>R- ACTGGTACAACGAGCGGATG	300 nM	60 ˚C
ACAA2	F- GGCTGTTGAGAGGAGTTTGG<br>R- GGCTGTGCTCTGAATGATGA	200 nM	60 ˚C
